# Anomalous celiac aorta supply to normal basal segments of the lung: a case report

**DOI:** 10.3389/fmed.2025.1655687

**Published:** 2025-10-03

**Authors:** Zhenhua Li, Rujuan Wang, Yiran Niu, Dongping Xia, Jixiang Ni

**Affiliations:** Department of Respiratory and Critical Care Medicine, The Central Hospital of Wuhan, Tongji Medical College, Huazhong University of Science and Technology, Wuhan, China

**Keywords:** anomalous celiac aorta supply to normal basal segment of the lung, hemoptysis, transarterial embolization, Amplatzer vascular plug, case report

## Abstract

We present a case of a 28-year-old female patient who has suffered from hemoptysis for a period of 10 years. Examinations revealed an anomalous celiac aorta supply to normal basal segment of the lung. The patient was improved after embolisation with a vascular plug. This case demonstrates the deadly risk posed by anomalous systemic arterial supply to normal basal segments of the lung. As a minimally invasive procedure, transarterial embolization (TAE) is a safe and effective therapeutic choice.

## Introduction

An anomalous celiac aorta supply to normal basal segment of the lung is an uncommon congenital anomaly. This condition is characterized by the presence of an anomalous artery originating from the celiac aorta, which supplies to normal basal lung segment. Patients frequently present with symptoms such as hemoptysis, chest discomfort, recurrent infections, or congestive heart failure secondary to pulmonary hyperperfusion or hypertension. The primary interventions employed in the management of this condition include surgical procedures and transarterial embolization (TAE). The present case report documents a successful management strategy for this vascular anomaly, employing the Amplatzer vascular plug (AVP) II embolization technique.

### Case description

**Figure 1:**
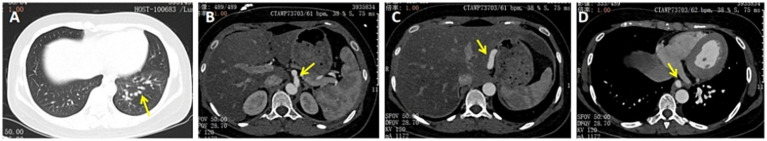
**(A)** CT of the chest showed thickening of the left lower pulmonary vein. **(B–D)** CTA showed bronchi in the blood supply area originating from the normal left lower pulmonary bronchi, and the pulmonary vein of the left lower hilar is thickened. The thick branch of the celiac aorta supplied part of the basal segments of the lung through the diaphragm (yellow arrow).

A 28-year-old female patient with a ten-year history of recurrent haemoptysis was transferred to our hospital. She had previously been diagnosed with bronchiectasis on multiple occasions at other medical facilities, despite undergoing treatment for both infection and haemostasis. She’s married with no children and no history of smoking. A physical examination revealed no abnormalities in her breathing or heart sounds. A comprehensive blood examination was conducted, encompassing various parameters including blood routine, liver and kidney function, and coagulation function. The results showed that the patient’s blood profile was normal. Computed tomography (CT) scan of the chest revealed thickening of the left lower pulmonary vein(Figure 1A). Subsequent computed tomography angiography (CTA) revealed an anomalous arterial branch arising from the celiac aorta,which originated at the T12-L1 level (Figures 1B–D). Angiographic examinations confirmed the presence of an anomalous artery originating from the celiac aorta. The diagnosis of anomalous celiac aorta supply to normal basal segments of the lung was thereby confirmed. Further diagnostic procedures were performed, including echocardiography. The results indicated a mean pulmonary artery pressure of 21 mmHg (normal range: 9–18 mmHg). There was no evidence of dilatation in the right atrium or right ventricle, nor of any observable enlargement of the pulmonary artery.

**Figure 2:**
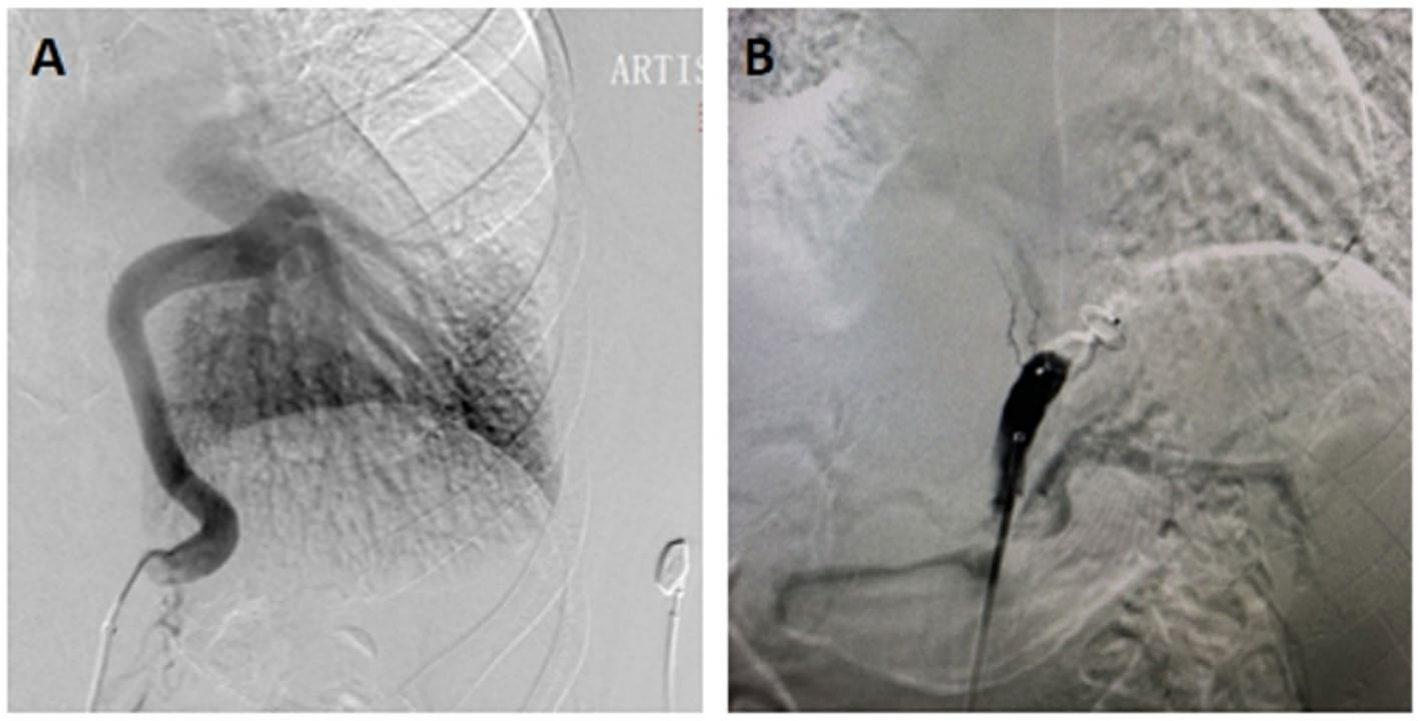
**(A)** Angiography showed a large supply arising from the celiac aorta to normal basal segments of the lung. **(B)** After proximal arterial embolization with AVP, a aortogram showed occlusion of the celiac aorta.

Following the presentation of treatment options, which included surgery, observation, or TAE, the patient opted for the latter due to its minimally invasive nature (Figure 2). Pre-procedural management encompassed a 3-day course of intravenous dexamethasone (5 mg daily) and antibiotics to prevent pulmonary infarction or infected necrosis. Subsequent to embolisation, the patient exhibited resolution of hemoptysis and chest discomfort, with discharge occurring on post-procedural day5.

## Discussion

Anomalous celiac aorta supply to normal basal segments of the lung was previously classified as type 1 pulmonary sequestration according to Pryce’s terminology since 1946 (1). This is a extremly rare congenital vascular malformation and most scholars consider this anomaly to differ from classic sequestration because of the normal bronchial connection (2). The subject is characterized by an anomalous systemic artery, drainage to the normal pulmonary vein, absence of the normal pulmonary artery, and no anomaly of the bronchus. The aetiology of this anomaly remains unclear, although it may be related to persistence of an embryonic connection between the aorta and pulmonary parenchyma (3).

Advances in imaging technology have enabled the definitive diagnosis of this anomaly through visualization of the pulmonary vasculature, bronchi, and parenchyma. Angiography further provides a haemodynamic assessment and evaluation of the shunt. This congenital malformation predominantly affects the left basal lung segment, typically manifesting as a solitary aberrant vessel originating from the left descending thoracic aorta. It is an uncommon occurrence for the congenital abnormality to be located in the right basal segment of the lung (4). In such cases, there is often an association with two or more anomalous arteries arising from either the proximal celiac aorta or the celiac trunk. Among the cases reported to date, only two were of this origin (5, 6). Our patient represents the third rare case. The most common clinical symptoms include hemoptysis, as was the case in the present instance. Furthermore, there have been reports of extensive hemoptysis resulting in death while the patient was under observation (7). In addition, there are literature reports on the clinical manifestations of dyspnoea, chest pain, recurrent infection and congestive heart failure (8). In our case, the patient presented with repeated episodes of hemoptysis and was misdiagnosed with bronchiectasis for a period of ten years. If not treated in a timely manner, this anomalous systemic artery-to-pulmonary vein drainage creates a left-to-left shunt, which can lead to progressive pulmonary hypertension, massive hemoptysis, and eventual heart failure. It is particularly pertinent to note that the likelihood of exacerbation of hemoptysis is increased during pregnancy (9).

The treatment of this abnormality is recommended for symptomatic or asymptomatic patients due to the risk of congestive heart failure in children and hemoptysis in adults (10). The main treatment has been surgical excision, such as lung lobectomy, ligation, or division of the anomalous artery. TAE is a procedure that shares similarities with surgical ligation as a treatment for this anomaly, yet it is a less invasive procedure. In recent years, an increasing number of cases have been selected for embolisation treatment (11–13). The necessity of endovascular treatment was determined as a means of averting the progression of pulmonary hypertension and the potential for sudden death from extensive hemoptysis. The utilisation of mechanical embolisation agents, encompassing coils, detachable balloons, Amplatzer occlusion devices and Amplatzer vascular plugs (AVPs), constitutes a viable approach for this purpose (14). However,the utilisation of liquid embolisation materials is not advised due to the elevated risk of extensive embolisation, which can potentially result in pulmonary embolisation and pulmonary infarction. Previous studies have demonstrated the efficacy of coils and AVPs in the embolisation of the ascending aorta. Jiang et al. conducted a comprehensive evaluation of various embolisation methodologies, juxtaposing the progression of embolisation techniques and embolisation agents through the course of time (14). The study concluded that the detachable AVP surpasses the utilisation of multiple coils in terms of efficacy for the embolisation of the ascending aorta. Consequently, this methodology was adopted, yielding positive outcomes; however, further observation is warranted to ensure long-term stability.

In summary, TAE represents a minimally invasive, safe, and effective approach for managing anomalous systemic arterial supply to pulmonary basal segments, with no major complications observed. It may thus serve as a primary alternative to surgical intervention. Periodic contrast-enhanced CT follow-up remains essential to monitor potential recanalization or hemoptysis recurrence from compensatory systemic collaterals.

## Data Availability

The original contributions presented in the study are included in the article/supplementary material, further inquiries can be directed to the corresponding author/s.
